# Ophthalmic Vascular Occlusion and Blindness After Platelet‐Rich Plasma Injections: A Systematic Review

**DOI:** 10.1111/jocd.70918

**Published:** 2026-06-07

**Authors:** Mandana Ebrahimzade, Shahriar Nazari, Mohammad Reza Pourani, Cristina Muñoz‐Gonzalez, Fahimeh Abdollahimajd, Maurizio Cavallini, Nabil Fakih‐Gomez

**Affiliations:** ^1^ Skin Research Center Shahid Beheshti University of Medical Sciences Tehran Iran; ^2^ Department of ENT and Head and Neck Surgery BMI Hospital Tehran Iran; ^3^ Private Practice. Zibaban Clinic, Management and Research of Complications Clinic Tehran Iran; ^4^ Private Practice. Facexpert Clinic and Academy Torremolinos Spain; ^5^ Clinical Research Development Unit Shohada‐e Tajrish Hospital, Shahid Beheshti University of Medical Sciences Tehran Iran; ^6^ Research Center of Artificial Intelligence in Health Shohada‐e Tajrish Hospital, Shahid Beheshti University of Medical Sciences Tehran Iran; ^7^ Agora Clinical Center Milan Italy; ^8^ Department of Facial Plastic & Cranio‐Maxillo‐Facial Surgery Fakih Hospital Khaizaran Lebanon

**Keywords:** aesthetic complications, blindness, ophthalmic ischemia, platelet‐rich plasma, retinal artery occlusion

## Abstract

**Background:**

Platelet‐rich plasma (PRP) is widely used in aesthetic and regenerative medicine; however, rare vision‐threatening complications such as blindness have been reported following facial injections.

**Aims:**

This systematic review aimed to identify and analyze all published cases of visual impairment following PRP injections.

**Methods:**

A systematic literature search was performed until August 2025, using PubMed, Embase, and Scopus, supplemented by a manual Google Scholar search. Search terms combined “platelet‐rich plasma” with ophthalmic complications. Case reports describing visual impairment after PRP injection were included without date or language restrictions. Data extracted included patient demographics, indication, injection site, onset and pattern of vision loss, ophthalmologic findings, management, and outcome.

**Results:**

Seven articles reporting 10 cases of PRP‐associated unilateral visual loss published between 2017 and 2025 were identified. All patients were female (22–64 years). Most cases (*n* = 9) followed PRP injections for facial rejuvenation, predominantly in the glabella (*n* = 6) and forehead (*n* = 4), while one followed scalp PRP for alopecia. Vision loss was immediate and painful in 9 cases; 8 presented with no light perception. Two cases with delayed or partial visual impairment demonstrated visual recovery after corticosteroid therapy. Fundoscopic findings indicated retinal and choroidal ischemia.

**Conclusions:**

Although rare, PRP injections can cause irreversible blindness, likely due to embolic ophthalmic artery occlusion (OAO). Prevention through safe injection technique is essential, as treatment options remain limited and often ineffective. Over half of the reported cases were performed by non‐physicians or inadequately trained practitioners, underscoring the critical need for proper credentials and training.

## 1 Introduction

Platelet‐rich plasma (PRP) is an autologous platelet concentrate obtained from peripheral blood via centrifugation in anticoagulant‐treated tubes. It contains a four‐ to seven‐fold increase in platelet concentration above baseline [1] and is rich in cytokines and growth factors released from α‐granules after activation by thrombin, calcium chloride, or collagen [2, 3]. Key growth factors include platelet‐derived growth factor (PDGF), vascular endothelial growth factor (VEGF), basic fibroblast growth factor (bFGF), epidermal growth factor (EGF), transforming growth factor β1 and β2 (TGF‐β1 and TGF‐β2), and insulin‐like growth factors 1 and 2 (IGF‐1 and IGF‐2), which regulate cell proliferation, differentiation, angiogenesis, and extracellular matrix (ECM) remodeling [4, 5]. Due to its regenerative properties, PRP has been widely adopted across numerous medical fields, ranging from aesthetic medicine to various therapeutic specialties [3, 5, 6, 7].

In aesthetics, PRP is used for wound repair, facial rejuvenation, and hair restoration. It stimulates fibroblasts and leukocyte‐derived MMP activity, promotes collagen and elastin synthesis, and increases hyaluronic acid (HA) production, improving dermal hydration and elasticity [5, 8]. Clinical studies report improvement in wrinkles, acne scars, skin texture, and periorbital pigmentation [2, 6, 9], although heterogeneity in protocols and outcome measures limits definitive conclusions on efficacy [4, 10]. PRP scalp mesotherapy has shown promising outcomes in androgenetic alopecia by stimulating mesenchymal stem cells, enhancing perifollicular angiogenesis, and promoting folliculogenesis [11, 12, 13, 14, 15, 16, 17]. Beyond aesthetics, intra‐articular PRP reduces inflammation and pain in temporomandibular joint disorders while stimulating cartilage regeneration [7, 18, 19].

PRP is cost‐effective, easy to prepare, and non‐immunogenic; however, risks include sterility issues, preparation errors, and variability in centrifugation protocols [3, 5, 20]. Despite its benefits, PRP has thrombogenic potential due to clotting factors and adhesive proteins [7, 21], and rare but severe complications have been reported, including irreversible blindness [3].

Vision‐threatening events occur after inadvertent intra‐arterial injection in high‐risk facial regions such as the glabella and nasal dorsum, where vessels anastomose with the ophthalmic artery (OA) [3, 22]. Retrograde embolization under high injection pressure may obstruct retinal circulation [22, 23], and as no reversal agent exists for PRP emboli, strict prevention is paramount.

This systematic review aims to provide a comprehensive overview of all published case reports of visual impairment following PRP injections worldwide, with the intention of increasing awareness among practitioners. A previous review published in 2022 identified seven such cases from four articles [24]. Given the rising use of PRP across multiple indications, we sought to update the literature and include any additional cases published in recent years, as the incidence of this rare but devastating complication may be increasing.

## 2 Methods

A systematic literature search was conducted until August 2025, in PubMed, Embase, and Scopus using a predefined search strategy that was adapted to each database, including the use of Medical Subject Headings (MeSH) in PubMed and Emtree explosion terms (/exp) in Embase. Google Scholar was also searched manually to identify additional records. The search terms included (“platelet‐rich plasma” OR PRP OR “platelet‐rich fibrin” OR PRF) AND (“blindness” OR “retinal artery occlusion” OR “retinal vein occlusion” OR “visual disorders” OR “ischemic optic neuropathy” OR “vision loss” OR “visual impairment” OR occlusion OR ischemia). No restrictions were applied regarding publication date or study design. After removal of duplicates, 1381 articles underwent title and abstract screening. Reference lists of all relevant articles and reviews were also screened to identify additional eligible cases. Full‐text review was performed for 12 articles that met the inclusion criterion of reporting a clinical case of visual impairment following PRP injection. Data extracted from each report included patient demographics, indication for PRP, injection site, time of onset and pattern of visual impairment, initial visual acuity (VA) and ophthalmological findings, associated symptoms, management strategies, and final visual outcome. The literature search and study selection were performed by M.E. and independently cross‐verified by M.R.P. to ensure methodological accuracy.

## 3 Results

**FIGURE 1:**
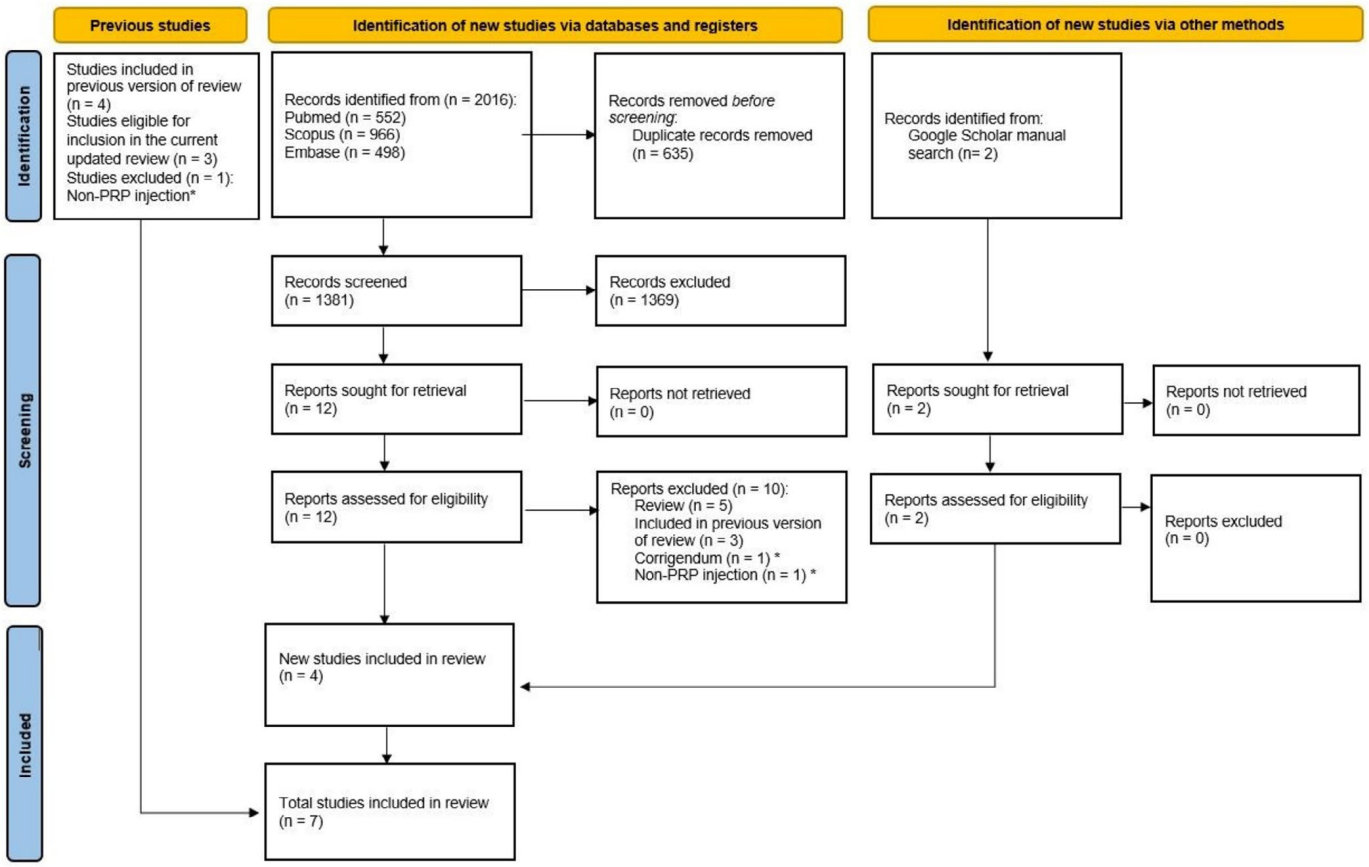
Flow diagram of the study selection process, following Preferred Reporting Items for Systematic Reviews and Meta‐Analyses (PRISMA) 2020 guidelines for updated systematic reviews, which included searches of databases, registers, and other sources. *Refers to a corrigendum to the 2020 Bhalla et al. article, published 2 years later in August 2022 (after the publication of the previous systematic review by Wu et al. in June 2022), declaring that “blindness by embolization was caused by activated mesenchymal pericyte plasma injections into TMJ, rather than PRP”. Hence, the 2020 Bhalla et al. article was no longer eligible for inclusion and was excluded with the label of “Non‐PRP injection”.

The systematic search identified 12 full‐text articles for eligibility assessment, of which five met the inclusion criteria. Three of these had been included in the previous systematic review by Wu et al. [25, 26, 27], and two were published later in 2023 and 2024 [21, 28]. A manual search in Google Scholar identified two additional eligible articles: one published in 2025 [29] and another from 2017 that had been missed in the earlier review [30]. The study by Bhalla et al. (2020) [31] was excluded because a corrigendum published in 2022 clarified that blindness had occurred following activated mesenchymal pericyte plasma rather than PRP injection [31]. In total, seven articles reporting 10 cases of unilateral visual impairment following PRP injection were included in the final analysis [21, 25, 26, 27, 28, 29, 30] (Table 1, Figure 1).

**TABLE 1:** Review of case reports on visual impairments after PRP injections.

Case No.	First author, year, country	Sex, age	Indication of PRP injection/injection site/injector	Vision loss side	Vision loss onset pattern	Associated symptoms	VA at initial evaluation	P/E findings	Fundoscopy findings of the affected eye	Imaging findings	Final diagnosis	Treatment	The longest follow‐up time	Final VA and outcome	Reversible/irreversible vision loss
1	Kalyam, 2017, USA [26]	F, 49	Facial skin Rejuvenation, rhytids correction/Bilateral glabella/ Unlicensed practitioner	R	Acute, sudden, immediately over the next few minutes of injection	R eye pain and fullness, severe nausea	At day 2: R: NLP L: 20/20	Pronounced R APD, restricted motility in supraduction and adduction (R exotropia and hypotropia in primary gaze), R moderate conjunctival hyperemia, Anterior segment: Unremarkable in both eyes, IOP: normal bilaterally, A 1 cm area of ecchymosis and induration above the R medial brow	At day 2: R: Profound optic disc pallor, diffuse retinal whitening including fovea, marked attenuation of arterioles with abrupt ending of the vessels in mid‐periphery, central macular edema, no cherry red spot (indicative of diffuse choroidal ischemia), no Hollenhorst plaque	At day 2: MRI/MRA: Restricted diffusion along the course of the R optic nerve, multiple subacute infarcts involving R frontal, parietal, and occipital lobes, Asymmetric abnormal FLAIR/T2 signal of the R medial rectus muscle suggestive of ischemia, Bone marrow edema within the R frontal bone with irregular enhancement involving the overlying skin Head and neck CT: R subacute frontal lobe ischemia without identifiable compromised vessels	Acute R OAO and brain infarction (R eye extraocular muscle ischemia and optic nerve infarction, along with R frontal, parietal, and occipital lobe infarction)	At day 2: (Outside the window of intra‐arterial tPA) Ocular massage, topical timolol 0.5%, brimonidine 0.2%, oral steroids, IV antibiotics	1 year	R: NLP/residual scarring and hard nodules of the R glabellar region (Ocular motility returned to normal by week 2)	Irreversible
2	Prado, 2017, USA [30]	F, 64	Facial skin Rejuvenation/Forehead/Family medicine clinician in a nail salon spa	R	Acute, sudden, immediately during injection	Severe R eye pain, syncope	At day 2 (24 h after symptom onset): R: NLP	Ischemic changes in the iris (suggestive of damage to posterior ciliary circulation, seen with very proximal OA occlusions), forehead necrosis in the distribution of the supraorbital artery, R eyelid ptosis, L hemiparesis	—	At day 2: MRI/MRA: Acute areas of infarct through much of the R middle cerebral artery territory, predominantly involving the superior R temporal lobe, the parietal and occipital lobes, and the cortex and subcortical white matter of the R frontal lobe (correlated with the contralateral L hemiparesis)	Proximal R OAO with distal changes associated with ischemia	At day 2: Antibiotic ointment for treatment of forehead necrosis followed by 3 treatments of pulsed dye laser to the scar after healing/Discharged with local wound care to the forehead and physical therapy to recover muscle tone from her CVA	8 months	No follow‐up VA/a slightly hypertrophic scar in the forehead necrosis area/some residual weakness of her L upper and lower extremity	Irreversible
3	Karam, 2020, Venezuela [25]	F, 61	Facial skin Rejuvenation/L glabella/Cosmetologist	L	Acute, sudden, immediately after injection	L eye pain, dizziness, vomiting	At day 1: R: 20/20 L: NLP	Glabellar bruising and hypoaesthesia in the distribution of the first trigeminal branch on the L side at day 3, followed by skin necrosis and ulceration of the injection area within a month	At day 1: L: Generalized retinal whitening, cherry‐red spot in the macula, segmental narrowing of the retinal arteries, and white material suggestive of an embolus within the CRA, attenuated retinal veins After 1 month: A pale optic disc with pigment in the superior temporal region, phantom retinal vessels, pigmentation of the peripheral retinal and macular fibrosis After 4 months: Increased pigmentation of the retina and optic disc (still pale)	At day 1: FA: patchy choroidal perfusion and blockage of the L retinal circulation Brain MRI/MRA: unremarkable At day 3: FA: limited and sluggish filling of the L retinal arteries and late hyperfluorescence in the retinal and perifoveal areas OCT: Increased L macular retinal thickness After 1 month: FA: Thinning of the retinal vessels and a hypofluorescent area mixed with mild areas of hyperfluorescence in the mid‐peripheral retinal and macular region OCT: Atrophy of all the retinal layers and fibrosis of the macular area	Iatrogenic L OAO, CRAO, and choroidal occlusion	—	8 months	No follow‐up VA/ L Retinal detachment, visible persistent scarring at the injection site	Irreversible
4	Karam, 2020, Venezuela [25]	F, 63	Facial skin Rejuvenation, rhytids correction/Forehead/Cosmetologist	R	Acute, sudden, immediately after injection	R eye pain, dizziness, tinnitus, vomiting, Iris depigmentation	At the first visit after 3 weeks of injection: R: NLP L: 20/20	Corneal endothelial pigmentation, iris atrophy, posterior synechiae of the iris and pigment dispersion at the anterior surface of the lens, glabellar scarring	At week 3: R: A pale R optic disc, CRA occlusion, and retinal hemorrhages, patchy pigment dispersion and macular fibrosis, Amalric triangle sign (indicative of choroidal Ischemia) in the mid‐peripheral retina	At week 3: FA: Delayed filling of the CRA, impaired perfusion of the optic disc and choroid, hyperfluorescence areas in the retina Brain MRI/MRA: unremarkable OCT: Fibrosis and neurosensory retinal detachment in the macular area with an epi‐retinal membrane	Iatrogenic L OAO, CRAO, and choroidal occlusion	—	No follow‐up	No follow‐up VA	Irreversible
5	Karam, 2020, Venezuela [25]	F, 52	Facial skin Rejuvenation/R nasolabial fold and glabella/Cosmetologist	R	Acute, sudden, immediately after injection	R eye pain, vomiting	At day 2 (24 h after injection): R: NLP L: 20/20	Incomplete R oculomotor nerve palsy (mild ptosis, restricted adduction and vertical gaze in R eye), flare in the anterior chamber and corneal folds, R eye IOP: 3 mmHg, bruising at the injection sites	At day 2: R: CRA occlusion without a cherry‐red spot but with multiple retinal hemorrhages	At day 2: FA: Delayed filling of the CRA and central retinal vein with areas of patchy choroidal non‐perfusion Brain MRI/MRA: unremarkable	Iatrogenic L OAO, CRAO, and choroidal occlusion	—	1 month	No follow‐up VA/ Necrosis of the forehead, R periorbital region, R cheek, and R nasal area	Irreversible
6	Karam, 2020, Venezuela [25]	F, 50	Facial skin Rejuvenation/Forehead, glabella, R eye external canthus/Cosmetologist	R	Acute, sudden, immediately after injection	R eye pain, transient blue vision, headache, nausea, urinary urgency, R ptosis	At the first visit after 3 weeks of injection: R: NLP L: 20/15	Complete R oculomotor nerve palsy	At week 3: R: Segmental narrowing of the retinal arteries, a pale retina with a CRA occlusion	At week 3: FA: Delayed filling of the central retinal artery with areas of patchy choroidal non‐perfusion	Iatrogenic L OAO, CRAO, and choroidal occlusion	—	No follow‐up	No follow‐up VA	Irreversible
7	Maslan, 2021, Malaysia [27]	F, 48	Facial skin Rejuvenation/Glabella/Aesthetic practitioner	L	Acute, sudden, immediately after injection	L ocular discomfort, occasional headache, numbness over the glabella site	At hour 9: R: 20/20 L: 20/60 At day 4: L: 10/200 (worsening of vision)	Grade 3 L relative APD, anterior segment: unremarkable L eye IOP: 16 mmHg, generalized constricted visual field in L eye At day 4: L periorbital hematoma and subconjunctival hemorrhage with 4+ anterior chamber cells	At day 1: L: Generalized edematous retina with dull foveal reflex but no cherry red spot At day 4: Hazy fundus but no vitritis At month 2: Pale and cupped optic disc with an increase cupping to 0.9	At day 1: OCTA: Significant reduction in both vessel and perfusion density of the superficial capillary plexus (SCP) in L eye Orbit MRI: Perineural enhancement of the L optic nerve MRV: Normal, no retro‐orbital hemorrhage	L CRAO and optic neuropathy	At hour 3: Ocular massage for 10–15 min, hyperventilation, IOP‐lowering oral medications At hour 9: Topical timolol 0.5%, topical dexamethasone 0.1%, and oral acetazolamide At day 4: Optic neuropathy treated with 3 days of IV methylprednisolone 250 mg QID followed by 11 days of oral prednisolone (1 mg/kg/d)	3 months	L: 20/20 (improved vision), remaining grade 2 L relative APD, decreased L red color saturation (8/10), significant improvement of L visual field, improvement in SCP perfusion and vessel density, pale optic disc with increased cupping to 0.9, resolved periorbital hematoma	Reversible
8	Iovino, 2023, Italy [28]	F, 45	Facial skin Rejuvenation/L glabella/—	L	Acute, sudden, immediately at the moment of the injection	L eye pain	At the first visit after 2 weeks of injection: R: 20/20 L: NLP	L anterior segment: unresponsive, fixed and dilated pupil, Normal L eye IOP (< 20 mmHg)	At week 2: L: Multiple patches of mild retinal whitening without a cherry‐red spot in the central macula, retinal vessels tortuosity with some pre‐retinal hemorrhages along the inferior temporal vascular arcade (in the mid and far peripheral retina), optic disk gliosis and boxcarring of the retinal vessels at the posterior pole Ultra‐wide field FAF: L: Diffuse and heterogeneous granular hyper/hypo autofluorescence	At week 2: Ultra‐wide field FA: Nearly absent retinal perfusion with multiple Amalric choroidal infarcts In either early and late‐stage FA phases in the L eye, in agreement with a critical non‐perfusion, an optic disc hyper fluorescence in the late‐phase Spectral‐domain OCT: A disorganization with a dramatic thinning of all retinal layers, scattered intra‐retinal hyperreflective foci, a pre‐retinal gliotic/fibrotic tissue around the optic disc of the L eye	Iatrogenic L OAO with profound ocular ischemia	Immediately: IV steroid (with no improvements in VA) SC Enoxaparin for 1 week (with no improvements in VA)	scheduled monthly follow‐up visits	L: NLP	Irreversible
9	Madala, 2024, USA [21]	F, 37	Facial skin Rejuvenation/L medial forehead/−	L	Acute, sudden, immediately seconds after injection	Bleeding, transient loss of consciousness, transient nausea, vomiting, difficulty speaking	At day 2: R: 20/20 L: NLP	Hypotonus L eye to palpation, significant relative APD by reverse in the L eye, profuse conjunctival chemosis and hyperemia, inferonasal subconjunctival hemorrhage, corneal edema with a diffuse haze and Descemet's folds, a heme‐filled anterior chamber in the L eye, L eye ptosis, limitations in supraduction, infraduction, abduction and adduction, irregular and nonreactive L pupil, R inferonasal subconjunctival hemorrhage	R: Retinal pigment epithelium degeneration along the retinal veins, nasal to the optic nerve FAF: R: Evidence of hypoautofluorescence suggestive of retinal atrophy as well as bordering hyperautofluorescence indicative of retinal pigment epithelium dysfunction (pigmented paravenous retinochoroidal atrophy nasally)	At day 2: Head CT with and without IV contrast: No acute cerebral ischemia or vascular occlusion FA: R: Marked choroidal hypoperfusion nasally	L entire orbital ischemia (both anterior and posterior segment ischemia) and eventual Phthisical eye; due to retrograde embolism to the OA and possibly smaller micro‐emboli occluding more distal branches of the OA	Planning for evisceration or enucleation with replacement with an ocular implant and prosthesis and orbital revolumization procedures	8 months	L: NLP/severe orbital volume loss, enophthalmos, a persistently edematous cornea with multiple folds and striae, and phthisis bulbi (Starting 6 months after the ischemic event)	Irreversible
10	Bibi, 2025, Pakistan [29]	F, 22	Hair restoration/scalp/−	L	Delayed, a month after injection	Non‐painful, black spots in the central eye field interspersed by grayish spots (central scotomas), reduced perception of brightness, bilateral transient blurring of vision 10 days post‐PRP that resolved within 2 days followed by the unilateral visual impairment a month after scalp PRP.	At the first visit after a month of injection: R: 6/9 L: 6/24	—	—	After a month of injection: OCT with RNLF: L: Macular edema and borderline thinning in the temporal sector of the retina (suggesting photoretinal layer disruption) Brain MRI: Pituitary microadenoma without compression of underlying optic chiasm (Non‐functional Pituitary incidentaloma)	Suspected and treated as a case of L optic neuropathy/May be explained by 1: Acute inflammatory macular neuroretinopathy (AMN) OR 2: Ischemic event or microvascular dysfunction	Initiated on a standard dose of Deltacortril (Prednisolone), starting with IV form (1 mg/kg/day) administered for 5 days, followed by oral maintenance Dose (0.5 mg/kg/day) for 3 weeks and tapering dose in fourth week	1 month	No follow‐up VA/Improved vision within 10 days of treatment, with complete resolution of visual loss within 1 month of treatment	Reversible

Abbreviations: APD, afferent pupillary defect; CRA, central retinal artery; CRAO, central retinal artery occlusion; CT, computed tomography; CVA, cerebrovascular accident; F, female; FA, fluorescein angiography; FAF, fundus autofluorescence; FLAIR, fluid‐attenuated inversion recovery; IOP, intraocular pressure; IV, intravenous; L, left; MRA, magnetic resonance angiography; MRI, magnetic resonance imaging; MRV, magnetic resonance venography; NLP, no light perception; OA, ophthalmic artery; OAO, ophthalmic artery occlusion; OCT, optical coherence tomography; OCTA, optical coherence tomography angiography; R, right; RNLF, retinal nerve fiber layer; SC, subcutaneous; tPA, tissue plasminogen activator; VA, visual acuity.

All affected patients were female, aged 22–64 years, originating from Venezuela (*n* = 4), USA (*n* = 3), Malaysia (*n* = 1), Italy (*n* = 1), and Pakistan (*n* = 1). Most cases (*n* = 9) occurred following PRP injection for facial rejuvenation, particularly for treatment of forehead lines and glabellar rhytids. One case (*n* = 1) followed scalp PRP treatment for hair loss [29].

The most common injection sites associated with visual impairment were the glabella (*n* = 6) and forehead (*n* = 4), followed by the nasolabial fold (*n* = 1) [25], lateral canthus (*n* = 1) [25], and scalp (*n* = 1) [29]. In several reports, PRP was injected into multiple facial areas during the same procedure. Injector qualification was not reported in three studies. In the remaining cases, injections were performed by cosmetologists/aesthetic practitioners (non‐physicians) (*n* = 5) [25, 27], an unlicensed practitioner (*n* = 1) [26], and a family physician (*n* = 1) [30].

All cases presented with unilateral visual loss, occurring ipsilateral to the injection site except in one delayed case following scalp PRP [29]. The onset was acute and painful in nine cases, beginning during or within minutes of injection, and delayed and painless in one patient [29]. Associated symptoms included periocular pain, headache, nausea, vomiting, dizziness, tinnitus, ptosis, ophthalmoplegia, visual field defects, and skin necrosis at the injection site.

Initial VA was no light perception (NLP) in eight of ten cases [21, 25, 26, 28, 30], indicating severe retinal and choroidal ischemia. Two patients had initially measurable VA and demonstrated partial or full recovery following treatment with corticosteroids [27, 29]. Presentation to medical care ranged from 3 h to 1 month after injection, with only two patients presenting within 24 h [25, 27].

Diagnostic findings included optic disc pallor, retinal whitening with or without cherry‐red spot, macular edema, and Amalric sign, suggesting choroidal ischemia. Fluorescein angiography showed delayed retinal artery filling and choroidal hypoperfusion, while optical coherence tomography revealed inner retinal atrophy and macular thinning. Two cases showed concomitant ischemic cerebral infarcts involving the middle cerebral artery territory on MRI [26, 30].

## 4 Discussion

Platelet‐rich plasma (PRP) is an autologous platelet concentrate derived from peripheral blood via centrifugation in anticoagulant‐treated tubes. PRP contains a platelet concentration four‐ to seven‐ fold above the physiological baseline (150000–400 000/μL) [1], along with a wide array of cytokines and growth factors. These bioactive molecules are released from α‐granules following platelet activation with agents such as thrombin, calcium chloride, or collagen [2, 3]. The principal growth factors in PRP include PDGF, VEGF, bFGF, EGF, TGF‐β1/β2, and IGF‐1/2. These mediators regulate cellular signaling pathways involved in stem cell stimulation, cell migration, proliferation, differentiation, angiogenesis, macrophage activation, and extracellular matrix (ECM) synthesis, thereby conferring PRP with significant pro‐regenerative and wound‐healing properties [4, 5]. Consequently, PRP has gained increasing attention in recent years as a promising therapeutic modality in dermatology, aesthetic medicine, plastic and maxillofacial surgery, dentistry, sports medicine, orthopedics, and other fields [3, 5, 6, 7].

In aesthetic and dermatologic practice, PRP is primarily used to promote wound healing, stimulate hair regrowth, and enhance facial rejuvenation as a minimally invasive anti‐aging treatment. It is hypothesized that PRP remodels damaged ECM by activating leukocyte‐derived matrix metalloproteinases (MMPs), promoting fibroblast migration and proliferation, and enhancing collagen and elastin production. These effects improve skin elasticity and firmness, while increased HA synthesis enhances hydration and dermal turgor [5, 8]. Clinical studies suggest that PRP improves skin texture and smoothness, reduces fine lines and wrinkles, and enhances the appearance of acne scars, nasolabial folds, and periorbital hyperpigmentation [2, 6, 9]. Nevertheless, due to heterogeneity in study designs and the lack of standardized treatment protocols and outcome measures, the overall clinical efficacy of PRP remains controversial [4, 10].

Over the past decade, intradermal PRP injections (mesotherapy) into the scalp have demonstrated encouraging results in the treatment of androgenetic alopecia in both men and women [11, 12, 13, 14, 15]. Acting as a reservoir of growth factors, cytokines, and plasma proteins, PRP exerts mitogenic effects on mesenchymal stem cells and stimulates fibroblasts, keratinocytes, endothelial cells, and smooth muscle cells. It enhances chemotaxis, mitogenesis, and ECM synthesis while promoting perifollicular angiogenesis and folliculogenesis to support hair restoration [12, 16, 17]. In addition to aesthetic applications, PRP has been used intra‐articularly for temporomandibular joint (TMJ) disorders. Its anti‐inflammatory and analgesic properties, along with the stimulation of chondrocytes and synoviocytes to regenerate cartilage and increase glycosaminoglycan production, have been associated with reduced pain and improved joint function [7, 18, 19].

PRP offers several practical advantages: it is relatively affordable, requires minimal equipment, and can be prepared quickly without the need for cell culture, allowing for immediate administration [3, 5]. While PRP is autologous and therefore non‐immunogenic, the procedure is not entirely risk‐free. Potential complications may arise from inadequate sterility, technical errors during preparation or injection, improper handling of PRP kits or centrifuges, variations in centrifugation protocols, or delays prior to injection [3, 20]. Furthermore, PRP possesses inherent thrombogenic properties due to its content of adhesive plasma proteins and clotting factors [7, 21]. Reported adverse effects range from local inflammation, tenderness, erythema, and edema to rare but devastating complications such as irreversible blindness [3].

Vision‐threatening complications in aesthetic practice result from inadvertent intra‐arterial injection of substances, such as dermal fillers, autologous fat, or PRP, within high‐risk facial regions, including the glabella, nasal dorsum, and nasolabial folds. These areas contain the supratrochlear, supraorbital, dorsal nasal, and angular arteries, which anastomose with the OA [3, 22]. Recent literature indicates a concerning rise in vision‐threatening complications from aesthetic filler injections. In a 2024 review article, a global total of 511 documented cases of filler‐induced blindness were reported up to March 2023 [32]. The vast majority of these cases were caused by HA and autologous fat fillers injected into high‐risk facial regions.

This updated systematic review identified 10 cases of PRP‐induced visual impairment worldwide, reaffirming that although the complication is rare, it is severe and potentially irreversible. Similar to previous findings in filler‐induced blindness, the glabellar region was the most common high‐risk injection site due to its vascular connection to the OA via the supratrochlear and supraorbital arteries. This mirrors prior evidence showing that glabellar and nasal injections account for most non‐filler ocular embolization events [1]. The primary underlying mechanism is explained by the retrograde flow of injected material through the supratrochlear, supraorbital, or dorsal nasal arteries toward the OA. This occurs when high injection pressure is applied to the syringe plunger, exceeding systolic arterial pressure and forcing the embolic material against normal blood flow. Higher pressures may propel the embolus beyond the origin of the OA, reaching the internal carotid system and potentially causing ischemic cerebrovascular accidents (CVA) and brain infarction, as demonstrated in cases 1 and 2 (Table 1).

Once the plunger pressure is released, systolic arterial pressure can drive the emboli anterogradely toward the distal branches of the OA, including the CRA, ciliary arteries, cilioretinal artery, branch retinal artery, and arterioles. Consequently, multifocal orbital ischemia may occur if the systolic pressure fragments the embolus into smaller microemboli that disseminate along the distal branches of the OA [33, 34]. This mechanism explains the simultaneous retinal and choroidal hypoperfusion observed in most cases (Cases: 1–6, 8, and 9; Table 1), sometimes accompanied by anterior chamber ischemia (Cases: 2, 4, 5, and 9; Table 1). The clinical manifestations and severity of symptoms depend on the site of arterial obstruction. Extensive occlusions, such as OAO, central retinal artery occlusion (CRAO), and generalized posterior ciliary artery occlusion (PCAO), result in complete vision loss (no light perception) due to retinal non‐perfusion. In contrast, branch retinal artery occlusion (BRAO) causes partial vision loss, while localized PCAO typically results in blurry vision [35]. Accordingly, the eight cases (Cases: 1–6, 8, and 9; Table 1) presenting with NLP at initial evaluation were ultimately diagnosed with OAO and subsequent CRAO.

The OAO is characterized by sudden, profound vision loss accompanied by severe ocular pain occurring immediately after injection, as reported in all eight OAO cases in this review [34]. Due to the proximal location of the arterial obstruction, most distal branches of the ophthalmic circulation are affected, predisposing to widespread orbital ischemia and multiple ophthalmic complications. These include ophthalmoplegia and ptosis resulting from ischemia of the extraocular muscles and their innervating cranial nerves (Cases: 1, 2, 5, 6, and 9; Table 1), and in more severe cases, iris atrophy and corneal edema secondary to anterior segment ischemia from long posterior ciliary artery occlusion (Cases: 2, 4, 5, and 9; Table 1) [34, 36]. Immediate onset of ocular pain following filler injection has been attributed to acute ischemia of the anterior segment and the levator palpebrae superioris muscle, and is considered a poor prognostic indicator for visual recovery in OAO [36]. Accordingly, the only painless case in our review (Case 10) demonstrated the most favorable outcome, achieving complete visual recovery. Furthermore, profound choroidal hypoperfusion, observed in all eight PRP‐induced OAO cases, has been previously identified as a strong predictor of poor visual prognosis in filler‐induced OAO [36, 37].

The cilioretinal artery, which originates from the choroidal circulation, is present in approximately 15%–20% of the population and may improve visual outcomes in retinal artery occlusion by providing dual arterial supply to the retina via both the CRA and the posterior ciliary circulation [27, 34]. Consequently, in cases of CRAO, central vision may be preserved if a cilioretinal artery is present. This anatomical variation likely explains why, in Case 7, despite the occurrence of CRAO, the patient did not experience complete vision loss, presumably due to preserved macular perfusion via the cilioretinal artery.

A key finding is that patients who developed complete and immediate vision loss had poor prognosis, likely due to proximal OAO, which results in irreversible ischemia within minutes. In contrast, delayed or partial visual loss may reflect optic neuropathy or distal microvascular occlusion, offering a short therapeutic window, as observed in the two cases that improved following systemic corticosteroid therapy [27, 29]. Therefore, distinguishing arterial occlusion from optic neuropathy at presentation is critical for prognosis and management.

Beyond anatomical considerations, the physicochemical properties of the injected material and the injection technique also influence the risk of vascular occlusion. Although PRP is autologous and lacks particulate filler components, its thrombogenic plasma proteins and fibrin matrix may still precipitate embolic phenomena or induce microvascular inflammation, ultimately resulting in retinal ischemia. The ischemic cascade is typically initiated by a primary embolic obstruction, followed by secondary inflammatory processes characterized by leukocyte adhesion, platelet aggregation, and activation of the coagulation cascade [33, 38]. In PRP‐induced vascular occlusion, this secondary phase may play a more significant role than the initial embolic event. Importantly, this process may be influenced by variation in PRP preparation techniques. Factors such as platelet and leukocyte concentration, platelet‐to‐leukocyte ratio, platelet‐to‐fibrin ratio, and the use of activation agents (e.g., calcium gluconate, calcium chloride, or thrombin) can alter the biological behavior and rheological properties of the final product, thereby affecting both safety and therapeutic outcomes [39, 40].

Currently, no standardized method for PRP preparation exists, and protocols are frequently modified by physicians based on clinical indication and desired regenerative effect [5, 39, 40, 41]. Notably, none of the 10 reported cases provided detailed information regarding PRP processing parameters or final composition. This lack of standardization highlights a critical knowledge gap regarding whether specific PRP formulations confer a higher risk of vascular occlusion.

Excessive injection force and velocity significantly increase intra‐arterial pressure during filler delivery [36]. Strong and sustained injection pressures (~166.7 mmHg) or the rapid administration of large bolus volumes (> 0.085 mL), particularly through large‐gauge needles, can exceed systolic arterial pressure. This allows retrograde migration of the injected material into the arterial system, especially within high‐risk anatomical regions referred to as facial “danger zones” [35]. Due to the retrospective nature of the case reports analyzed, detailed information regarding injection techniques was not provided in any of the 10 cases, making it impossible to determine the extent to which technical factors contributed to vascular occlusion. However, technical error cannot be excluded, particularly given the lack of information regarding injector qualifications in nine cases. Only one case (Case 1) specified injector credentials, revealing that the procedure had been performed by an unlicensed practitioner [26]. The designation “cosmetologist” or “aesthetic practitioner,” reported in five of the ten cases, is insufficient to determine medical training, licensure, or procedural competency [20]. Notably, a substantial proportion of procedures were performed by non‐physicians or inadequately trained personnel, with unreported injector qualifications. This raises serious concerns regarding patient safety, particularly in high‐risk anatomical regions such as the glabella and forehead, as the lack of medical training and detailed knowledge of facial vascular anatomy likely contributed to inadvertent intra‐arterial injection in high‐risk zones.

### 4.1 Clinical Implications

#### 4.1.1 Prevention

According to the findings of this review, prevention remains the most effective strategy to avoid PRP‐induced vascular complications, as no pharmacologic agent exists to reverse PRP embolization. A thorough knowledge of facial vascular anatomy, with particular emphasis on high‐risk zones including the glabella, forehead, nasal dorsum, and nasolabial folds, followed by proper hands‐on training in safe injection techniques (such as minimal and controlled pressure on the plunger, slow velocity of injection, small volumes of injection per bolus, aspiration prior to injection, and choosing an appropriate gauge of cannula/needle (25G or greater bore)) with preferential use of blunt cannulas, is essential to minimize the risk of catastrophic complications such as OAO [42]. The establishment of standardized certification programs for aesthetic practitioners is necessary to reduce preventable adverse outcomes, as it allows for procedures to be ideally performed by qualified medical professionals with formal training and accreditation in aesthetic injections.

Furthermore, patient selection and informed consent are critical, with explicit discussion of rare but severe complications such as blindness. Precise cautions should be taken regarding PRP absolute and relative contraindications prior to administration [1]. Particular cautions should be taken for medical conditions imposing a hypercoagulative state on patients, such as systemic corticosteroids, tobacco, or oral contraceptive use, as well as recent illnesses and fevers. Precise history taking and medical reconciliation over general medical health of patients, not only on cosmetic expectations, helps to figure out high‐risk patients requiring closer observations and extended follow‐up [26].

#### 4.1.2 Proposed Complication Management

Although definitive evidence‐based guidelines cannot be established from this review alone, due to the limited data and retrospective nature of the case reports, the following pragmatic approach is proposed for clinicians based on both the patterns observed from the cases and available literature [35, 38, 43, 44, 45, 46]:

1. Immediate recognition and referral.

Management of acute visual loss after aesthetic injections requires prompt diagnosis and interventions. Any acute visual symptoms (e.g., pain, vision change, ptosis, and ophthalmoplegia) during or immediately after facial/scalp PRP injection should be treated as an ophthalmologic emergency requiring immediate referral to an ophthalmologic center within a 15–90‐min window, while the still‐viable retinal tissue is salvageable. Reported therapeutic windows in the literature vary widely, ranging from as short as 15 min to as long as 90–240 min [35].

2 Stop injection immediately.
3 Initial supportive measures (to dilate the retinal arteries and decrease the IOP while arranging transfer).
  - Ocular massage for 10–15 min (repeating sets of 5 s pressure, 10 s release).
  - Placing the patient in a supine position with a 15–45 degrees' head rise.
  - IOP‐lowering agents (e.g., topical timolol and oral acetazolamide).
  - Anterior chamber paracentesis if feasible.
  - Carbon therapy and/or hyperventilation (e.g., rebreathing into a paper bag for approximately 10 min every half an hour).

Lower IOP may help with dislodging the embolus into more distal and peripheral retinal vessels, preserving the central vision [35]. However, these emergency management strategies are often unsuccessful when treatment is delayed beyond 90 min.

4 Pharmacologic considerations.

As no pharmacologic agent exists to exclusively reverse PRP embolization. Unlike filler embolism, hyaluronidase has no therapeutic role in PRP‐induced occlusion. There is no specific reversal agent to erase the PRP‐induced embolus unless anticoagulation by heparinization or intravenous or intra‐arterial thrombolytic treatments are applied to prevent or lysis of further thrombosis [38].

Systemic corticosteroids (e.g., intravenous methylprednisolone followed by oral maintenance and taper) may be beneficial in cases of partial/delayed vision loss or suspected optic neuropathy/inflammatory component, as seen in the two reversible cases [27, 29].

Regarding a recent publication, we suggest that injection of botulinum toxin (BoNT) may be beneficial in this rare occurrence because of its vasodilatory effect [47]. Moreover, the risks and benefits of a fixed eye following BoNT treatment should be carefully assessed; however, it might prove advantageous in this emergency complication.

5 Monitoring and support.

- Screening for associated cerebral ischemia (MRI/MRA when concomitant CVA is suspected).
- Providing supportive care for skin necrosis (topical or IV antibiotics followed by scar healing treatments).
- Long‐term follow‐up for phthisis bulbi or secondary complications. Planning for evisceration or enucleation with replacement with an ocular implant and prosthesis and orbital may be the next step [21].

It seems the swift diagnosis and management of ischemic ocular complications promptly during or following PRP is a key takeaway hint; particularly in cases of partial vision loss. All physicians should be vigilant during injection in high‐risk anatomic sites, and, following the first ocular symptom, without hesitation, initiate the main procedures for decreasing ocular pressure and possibly resolving vascular occlusion [48].

### 4.2 Limitations and Future Directions

This review is limited by the small number of published cases and the inherent constraints of case‐report‐based evidence, including incomplete clinical documentation, potential publication bias, and lack of standardized diagnostic or treatment protocols. Data synthesis was not feasible due to the qualitative nature of the included studies.

Heterogeneity in PRP preparation methods, platelet concentration, activation status, and injection techniques across reports also limits the ability to establish causality or estimate true incidence. Future research should prioritize the creation of international registries for vascular complications associated with injectable treatments, including PRP. Experimental and clinical studies are also needed to elucidate the underlying pathophysiology of PRP‐induced vascular events and to evaluate potential neuroprotective and reperfusion strategies.

Importantly, given that over half of the reported cases were performed by non‐physician or unlicensed injectors, future directions must emphasize mandatory, structured training courses for all practitioners performing PRP aesthetic injections which could help mitigate risks associated with inadequate credentials. Finally, the development of consensus‐based safety guidelines and more evidence‐based complication management algorithms specific to PRP is essential to improve patient safety in aesthetic practice.

## 5 Conclusion

Visual impairment following PRP injection is a rare but catastrophic complication, most often irreversible and associated with injections in high‐risk facial vascular zones. This systematic review identified 10 published cases worldwide, the majority presenting with sudden vision loss from presumed OAO. Despite being autologous, PRP can cause serious vascular events similar to dermal fillers. Prevention through safe injection techniques, anatomical awareness, and avoidance of high‐risk sites is essential, along with rapid recognition of ocular symptoms and urgent referral, as the treatment window is extremely limited. Standardized evidence‐based safety guidelines and practitioner training are urgently needed.

## Author Contributions

M.E.: Data curation, formal analysis, validation, writing – original draft, and writing – review and editing; S.N.: Review and editing, supervision; M.R.P.: Conceptualization, data curation, methodology, writing – original draft, and writing – review and editing; C.M.‐G.: Review and editing, supervision; F.A.: Conceptualization, methodology, writing – review and editing, and supervision; M.C.: Review and editing, supervision; N.F.‐G.: Conceptualization, methodology, writing – review and editing, and supervision.

## Funding

The authors have nothing to report.

## Ethics Statement

The authors have nothing to report.

## Consent

The authors have nothing to report.

## Conflicts of Interest

The author Nabil Fakih‐Gomez is consultant for Merz Aesthetics (Frankfurt, Germany).

## Data Availability

Data sharing not applicable to this article as no datasets were generated or analysed during the current study.

## References

[1] G. L. Peng , “Platelet‐Rich Plasma for Skin Rejuvenation: Facts, Fiction, and Pearls for Practice,” Facial Plastic Surgery Clinics of North America 27, no. 3 (2019): 405–411, 10.1016/j.fsc.2019.04.006. 31280855

[2] P. Gentile and S. Garcovich , “Systematic Review: Platelet‐Rich Plasma Use in Facial Rejuvenation,” Plastic and Reconstructive Surgery 152, no. 1 (2023): 72e–82e, 10.1097/PRS.0000000000010150. 36728559

[3] A. Arita and M. Tobita , “Adverse Events Related to Platelet‐Rich Plasma Therapy and Future Issues to Be Resolved,” Regenerative Theraphy 20, no. 26 (2024): 496–501, 10.1016/j.reth.2024.07.004. PMC11295534 39100535

[4] H. Xiao , D. Xu , R. Mao , M. Xiao , Y. Fang , and Y. Liu , “Platelet‐Rich Plasma in Facial Rejuvenation: A Systematic Appraisal of the Available Clinical Evidence,” Clinical, Cosmetic and Investigational Dermatology 16, no. 14 (2021): 1697–1724, 10.2147/CCID.S340434. PMC8606573 34819739

[5] P. Samadi , M. Sheykhhasan , and H. M. Khoshinani , “The Use of Platelet‐Rich Plasma in Aesthetic and Regenerative Medicine: A Comprehensive Review,” Aesthetic Plastic Surgery 43, no. 3 (2019): 803–814, 10.1007/s00266-018-1293-9. 30552470

[6] M. Neiva‐Sousa , C. Carracha , L. Nunes da Silva , and P. Valejo Coelho , “Does Platelet‐Rich Plasma Promote Facial Rejuvenation? Revising the Latest Evidence in a Narrative Review,” Journal of Cutaneous and Aesthetic Surgery 16, no. 4 (2023): 263–269, 10.4103/JCAS.JCAS_210_22. 38314356 PMC10833488

[7] C. Haddad , A. Zoghbi , E. El Skaff , and J. Touma , “Platelet‐Rich Plasma Injections for the Treatment of Temporomandibular Joint Disorders: A Systematic Review,” Journal of Oral Rehabilitation 50, no. 11 (2023): 1330–1339, 10.1111/joor.13545. 37341166

[8] B. Hersant , M. SidAhmed‐Mezi , C. Aboud , et al., “Synergistic Effects of Autologous Platelet‐Rich Plasma and Hyaluronic Acid Injections on Facial Skin Rejuvenation,” Aesthetic Surgery Journal 41, no. 7 (2021): NP854–NP865, 10.1093/asj/sjab061. 33534905

[9] M. Banihashemi , N. Zabolinejad , M. Salehi , D. Hamidi Alamdari , and S. Nakhaizadeh , “Platelet‐Rich Plasma Use for Facial Rejuvenation: A Clinical Trial and Review of Current Literature,” Acta Biologica et Medica 92, no. 2 (2021): e2021187, 10.23750/abm.v92i2.9687. PMC8182581 33988167

[10] B. Atiyeh , A. Oneisi , and F. Ghieh , “Platelet‐Rich Plasma Facial Rejuvenation: Myth or Reality?,” Aesthetic Plastic Surgery 45, no. 6 (2021): 2928–2938, 10.1007/s00266-021-02300-9. 33999221

[11] G. Schiavone , D. Raskovic , J. Greco , and D. Abeni , “Platelet‐Rich Plasma for Androgenetic Alopecia: A Pilot Study,” Dermatologic Surgery 40, no. 9 (2014): 1010–1019, 10.1097/01.DSS.0000452629.76339.2b. 25111436

[12] P. Gentile and S. Garcovich , “Systematic Review of Platelet‐Rich Plasma Use in Androgenetic Alopecia Compared With Minoxidil, Finasteride, and Adult Stem Cell‐Based Therapy,” International Journal of Molecular Sciences 21, no. 8 (2020): 2702, 10.3390/ijms21082702. 32295047 PMC7216252

[13] N. L. Tamashunas and W. F. Bergfeld , “Male and Female Pattern Hair Loss: Treatable and Worth Treating,” Cleveland Clinic Journal of Medicine 88, no. 3 (2021): 173–182, 10.3949/ccjm.88a.20014. 33648970

[14] A. F. Q. de Oliveira , F. P. N. Arcanjo , M. R. P. Rodrigues , A. A. Rosa E Silva , and P. R. Hall , “Use of Autologous Platelet‐Rich Plasma in Androgenetic Alopecia in Women: A Systematic Review and Meta‐Analysis,” Journal of Dermatological Treatment 34, no. 1 (2023): 2138692, 10.1080/09546634.2022.2138692. 36264022

[15] M. Roohaninasab , A. Goodarzi , M. Ghassemi , A. Sadeghzadeh‐Bazargan , E. Behrangi , and N. Najar Nobari , “Systematic Review of Platelet‐Rich Plasma in Treating Alopecia: Focusing on Efficacy, Safety, and Therapeutic Durability,” Dermatologic Therapy 34, no. 2 (2021): e14768, 10.1111/dth.14768. 33421285

[16] B. Singh and L. J. Goldberg , “Autologous Platelet‐Rich Plasma for the Treatment of Pattern Hair Loss,” American Journal of Clinical Dermatology 17, no. 4 (2016): 359–367, 10.1007/s40257-016-0196-2. 27234711

[17] R. Alves and R. Grimalt , “A Review of Platelet‐Rich Plasma: History, Biology, Mechanism of Action, and Classification,” Skin Appendage Disorders 4, no. 1 (2018): 18–24, 10.1159/000477353. 29457008 PMC5806188

[18] B. M. Sousa , N. López‐Valverde , A. López‐Valverde , et al., “Different Treatments in Patients With Temporomandibular Joint Disorders: A Comparative Randomized Study,” Medicina (Kaunas, Lithuania) 56, no. 3 (2020): 113, 10.3390/medicina56030113. 32151101 PMC7142788

[19] J. Xu , H. Ren , S. Zhao , et al., “Comparative Effectiveness of Hyaluronic Acid, Platelet‐Rich Plasma, and Platelet‐Rich Fibrin in Treating Temporomandibular Disorders: A Systematic Review and Network Meta‐Analysis,” Head & Face Medicine 19, no. 1 (2023): 39, 10.1186/s13005-023-00369-y. 37633896 PMC10463486

[20] C. McGloin , “Platelet‐Rich Plasma: How Safe Is It, and Can It Cause Irreversible Blindness?,” Journal of Aesthetic Nursing 11 (2022): 154–162, 10.12968/joan.2022.11.4.154.

[21] S. Madala , Y. K. Bao , J. H. Lee , J. Gluckstein , J. Chang , and S. Zhang‐Nunes , “Phthisical Eye and Orbital Ischemia After Cosmetic Platelet‐Rich Plasma Injection to the Forehead,” American Journal of Ophthalmology Case Reports 34 (2023): 101968, 10.1016/j.ajoc.2023.101968. 38601194 PMC11004058

[22] L. De‐Pablo‐Gómez‐de‐Liaño , F. Ly‐Yang , B. Burgos‐Blasco , and J. I. Fernández‐Vigo , “Ophthalmological Complications of Aesthetic Medicine Procedures: A Narrative Review,” Journal of Clinical Medicine 14, no. 15 (2025): 5399, 10.3390/jcm14155399. 40807018 PMC12347381

[23] K. Beleznay , J. D. A. Carruthers , S. Humphrey , A. Carruthers , and D. Jones , “Update on Avoiding and Treating Blindness From Fillers: A Recent Review of the World Literature,” Aesthetic Surgery Journal 39, no. 6 (2019): 662–674, 10.1093/asj/sjz053. 30805636

[24] S. Z. Wu , X. He , and R. A. Weiss , “Vision Loss After Platelet‐Rich Plasma Injection: A Systematic Review,” Dermatologic Surgery 48, no. 6 (2022): 697–698, 10.1097/DSS.0000000000003451. 35412481

[25] E. Z. Karam , A. Gan , R. Muci Mendoza , E. Martinez , and E. Perez , “Visual Loss After Platelet‐Rich Plasma Injection Into the Face,” Neuro‐Ophthalmology 44, no. 6 (2020): 371–378, 10.1080/01658107.2020.1740936. 33335344 PMC7722697

[26] K. Kalyam , S. C. Kavoussi , M. Ehrlich , et al., “Irreversible Blindness Following Periocular Autologous Platelet‐Rich Plasma Skin Rejuvenation Treatment,” Ophthalmic Plastic and Reconstructive Surgery 33, no. 3S Suppl 1 (2017): S12–S16, 10.1097/IOP.0000000000000680. 27015236

[27] N. Maslan , W. H. Wan Abdul Halim , N. M. Din , and S. F. Tang , “Central Retinal Artery Occlusion and Optic Neuropathy Secondary to Platelet Rich Plasma Injection: A Case Report,” International Journal of Ophthalmology 14, no. 6 (2021): 945–947, 10.18240/ijo.2021.06.23. 34150553 PMC8165622

[28] C. Iovino , F. Testa , L. Cristiano , L. De Rosa , G. De Rosa , and F. Simonelli , “Iatrogenic Ophthalmic Artery Occlusion After Platelet‐Rich Plasma Dermal Filler Documented With Ultra‐Widefield Imaging,” European Journal of Ophthalmology 33, no. 6 (2023): NP74–NP78, 10.1177/11206721231156635. 36803055

[29] F. Bibi , A. Sanan , M. Hamza , et al., “Reversible Vision Loss With Pituitary Microadenoma Following PRP Injection for Hair Growth: A Rare Case Report and a Mini‐Review of the Literature,” Clinical Case Reports 13, no. 10 (2025): e71191, 10.1002/ccr3.71191. 41069749 PMC12504142

[30] G. Prado and J. Rodríguez‐Feliz , “Ocular Pain and Impending Blindness During Facial Cosmetic Injections: Is Your Office Prepared?,” Aesthetic Plastic Surgery 41, no. 1 (2017): 199–203, 10.1007/s00266-016-0728-4. 28032150

[31] M. Bhalla , Z. El‐Housseini , and R. Asaria , “Blindness Associated With Platelet‐Rich Plasma Temporomandibular Joint Injections,” British Journal of Oral & Maxillofacial Surgery 58, no. 9 (2020): 1197–1199, 10.1016/j.bjoms.2020.08.042. 32928585

[32] V. C. Doyon , C. Liu , R. Fitzgerald , et al., “Update on Blindness From Filler: Review of Prognostic Factors, Management Approaches, and a Century of Published Cases,” Aesthetic Surgery Journal 44, no. 10 (2024): 1091–1104, 10.1093/asj/sjae091. 38630871

[33] S. Madala , J. Li , J. Gluckstein , and S. Zhang‐Nunes , “Management of Visual Complications of Dermal Filler Injections,” Plastic and Aesthetic Research 11, no. 10 (2024), 10.20517/2347-9264.2023.100.

[34] J. S. Lee , J. Y. Kim , C. Jung , and S. J. Woo , “Iatrogenic Ophthalmic Artery Occlusion and Retinal Artery Occlusion,” Progress in Retinal and Eye Research 78 (2020): 100848, 10.1016/j.preteyeres.2020.100848. 32165219

[35] L. Walker , C. Convery , E. Davies , G. Murray , and B. Croasdell , “Consensus Opinion for the Management of Soft Tissue Filler Induced Vision Loss,” Journal of Clinical and Aesthetic Dermatology 14, no. 12 (2021): E84–E94. 35096260 PMC8794490

[36] S. W. Park , S. J. Woo , K. H. Park , J. W. Huh , C. Jung , and O. K. Kwon , “Iatrogenic Retinal Artery Occlusion Caused by Cosmetic Facial Filler Injections,” American Journal of Ophthalmology 154, no. 4 (2012): 653–662.e1, 10.1016/j.ajo.2012.04.019. 22835509

[37] V. Chatrath , P. S. Banerjee , G. J. Goodman , and E. Rahman , “Soft‐Tissue Filler‐Associated Blindness: A Systematic Review of Case Reports and Case Series,” Plastic and Reconstructive Surgery. Global Open 7, no. 4 (2019): e2173, 10.1097/GOX.0000000000002173. 31321177 PMC6554164

[38] S. Madala , S. Davuluru , J. Li , et al., “Management of Vision Loss Associated With Complications of Cosmetic Filler Injections,” Frontiers in Ophthalmology 5 (2025): 1568370, 10.3389/fopht.2025.1568370. 40291837 PMC12021870

[39] R. Pensato , R. Al‐Amer , and S. La Padula , “Ocular Pain and Impending Blindness During Facial Cosmetic Injections: Is Your Office Prepared?,” Aesthetic Plastic Surgery 48, no. 15 (2024): 3034–3035, 10.1007/s00266-023-03588-5. 37605019

[40] S. Dashore , K. Chouhan , S. Nanda , and A. Sharma , “Preparation of Platelet‐Rich Plasma: National IADVL PRP Taskforce Recommendations,” Indian Dermatology Online Journal 12, no. Suppl 1 (2021): S12–S23, 10.4103/idoj.idoj_269_21. 34976877 PMC8664176

[41] P. Everts , K. Onishi , P. Jayaram , J. F. Lana , and K. Mautner , “Platelet‐Rich Plasma: New Performance Understandings and Therapeutic Considerations in 2020,” International Journal of Molecular Sciences 21, no. 20 (2020): 7794, 10.3390/ijms21207794. 33096812 PMC7589810

[42] M. D. Humzah , S. Ataullah , C. Chiang , R. Malhotra , and R. Goldberg , “The Treatment of Hyaluronic Acid Aesthetic Interventional Induced Visual Loss (AIIVL): A Consensus on Practical Guidance,” Journal of Cosmetic Dermatology 18, no. 1 (2019): 71–76, 10.1111/jocd.12672. 29885087 PMC6912247

[43] S. S. Hayreh , M. B. Zimmerman , A. Kimura , and A. Sanon , “Central Retinal Artery Occlusion. Retinal Survival Time,” Experimental Eye Research 78, no. 3 (2004): 723–736, 10.1016/s0014-4835(03)00214-8. 15106952

[44] S. Tobalem , J. S. Schutz , and A. Chronopoulos , “Central Retinal Artery Occlusion—Rethinking Retinal Survival Time,” BMC Ophthalmology 18, no. 1 (2018): 101, 10.1186/s12886-018-0768-4. 29669523 PMC5907384

[45] S. S. Hayreh , “Central Retinal Artery Occlusion,” Indian Journal of Ophthalmology 66, no. 12 (2018): 1684–1694, 10.4103/ijo.IJO_1446_18. 30451166 PMC6256872

[46] C. DeSai and A. Hays Shapshak , “Cerebral Ischemia,” in StatPearls [Internet] (StatPearls Publishing, 2023). 32809345

[47] S. Nazari , N. Fakih‐Gomez , N. Hadadian , et al., “A New Protocol (THIS and FAT) for the Treatment of Filler‐Induced Vascular Occlusion: A Case Series,” Frontiers in Medicine 12 (2025): 1585983. 40809419 10.3389/fmed.2025.1585983 PMC12343624

[48] S. Barbarino , S. Khalifian , and J. Fezza , “EYE‐CODE Protocol for the Nonophthalmologist for Treatment of Retinal Artery Occlusion After Intra‐Arterial Injection of Soft‐Tissue Fillers: 2025 Update,” Journal of Cosmetic Dermatology 24, no. 7 (2025): e70336, 10.1111/jocd.70336. 40631662 PMC12239549

